# Synthesis, Characterization and In Vitro Evaluation of Chitosan Nanoparticles Physically Admixed with Lactose Microspheres for Pulmonary Delivery of Montelukast

**DOI:** 10.3390/polym14173564

**Published:** 2022-08-29

**Authors:** Faqir Ullah, Kifayat Ullah Shah, Shefaat Ullah Shah, Asif Nawaz, Touseef Nawaz, Kamran Ahmad Khan, Raed F. Alserihi, Hossam H. Tayeb, Shams Tabrez, Mulham Alfatama

**Affiliations:** 1 Faculty of Pharmacy, Gomal University, Dera Ismail Khan 29050, Pakistan; 2 Department of Medical Laboratory Sciences, Faculty of Applied Medical Sciences, King Abdulaziz University, Jeddah 21589, Saudi Arabia; 3 King Fahd Medical Research Center, King Abdulaziz University, Jeddah 21589, Saudi Arabia; 4 Nanomedicine Unit, Center of Innovation in Personalized Medicine, King Abdulaziz University, Jeddah 21589, Saudi Arabia; 5 Faculty of Pharmacy, Universiti Sultan Zainal Abidin, Besut Campus, Besut 22200, Terengganu, Malaysia

**Keywords:** montelukast, chitosan, lactose, nanoparticles, microspheres, inhalation drug delivery

## Abstract

This study aimed to synthesise montelukast-loaded polymeric nanoparticles via the ionic gelation method using chitosan as a natural polymer and tripolyphosphate as a crosslinking agent. Tween 80, hyaluronic acid and leucine were added to modify the physicochemical properties of nanoparticles, reduce the nanoparticles’ uptake by alveolar macrophages and improve powder aerosolisation, respectively. The nanoparticles ranged from 220 nm to 383 nm with a polydispersity index of ≤0.50. The zeta potential of nanoparticles ranged from 11 mV to 22 mV, with a drug association efficiency of 46–86%. The simple chitosan nanoparticles (F2) were more spherical in comparison to other formulations (F4–F6), while the roughness of hyaluronic acid (F5) and leucine (F6) added formulations was significantly high er than F2 and Tween 80 added formulation (F4). The DSC and FTIR analysis depict that the physical and chemical properties of the drug were preserved. The release of the drugs from nanoparticles was more sustained in the case of F5 and F6 when compared to F2 and F4 due to the additional coating of hyaluronic acid and leucine. The nanoparticles were amorphous and cohesive and prone to exhalation due to their small size. Therefore, nanoparticles were admixed with lactose microspheres to reduce particle agglomeration and improve powder dispersion from a dry powder inhaler (DPI). The DPI formulations achieved a dispersed fraction of 75 to 90%, a mass median aerodynamic diameter (MMAD) of 1–2 µm and a fine particle fraction (FPF) of 28–83% when evaluated using the Anderson cascade impactor from Handihaler^®^. Overall, the montelukast-loaded nanoparticles physically admixed with lactose microspheres achieved optimum deposition in the deep lung for potential application in asthmatic patients.

## 1. Introduction

The respiratory system is one of the most important organs in the body for systemic and/or local medication administration [1]. Treatment for two lung conditions, including asthma and chronic obstructive pulmonary disease (COPD), relies heavily on oral inhalation of medications [2]. Chronic inflammatory disease of the respiratory system, asthma, is characterised by airway inflammation. Approximately 334 million children between the ages of 10 and 14 and seniors between the ages of 75 and 79 are affected globally [3]. The combination of inflammatory mediators, such as cytokines and leukotrienes (LTs), and environmental variables play a significant role in the pathogenesis of asthma. By increasing the vascular permeability of the lung airways, cysteinyl LTs (CysLTs) can cause pulmonary oedema and, as a result, mucus hypersecretion [4]. Consequently, the air entering the lungs is reduced in asthmatic patients due to their enlarged, sensitive and inflamed airways [5].

Montelukast (R,E)-2-(1-((1-(3-(2-(7-chloroquinolin-2-yl) vinyl) phenyl), a powerful and specialised antagonist of the cysteinyl leukotriene receptor 1 subtype (CysLT1), is 3-(2-(2-hydroxypropan-2-yl) phenyl) propylthio) methyl) cyclopropyl) acetic acid [6]. Montelukast is a medication frequently used in the form of oral tablets and granules for the prevention and treatment of asthma because it can effectively counteract the negative effects of leukotriene D4-mediated CysLT1-receptor activation on the lung’s airway [7]. However, oral montelukast has a considerable first-pass metabolism and is widely distributed in the peripheral compartments [8]. The hepatic metabolism of CYP2C8 and transporter-mediated uptake result in pharmacokinetic variability and drug–drug interactions [9].

Pulmonary drug delivery provides a non-invasive method for the site-specific treatment of chronic respiratory disorders [10]. Due to a lack of dependable delivery vehicles, few medicines delivered via the pulmonary route have reached the clinical phase [11]. Because of the distinct characteristics of the human respiratory tract and lung microenvironment, the pulmonary delivery system design had to adhere to stricter constraints than other focused delivery systems. The pulmonary clearance processes, such as respiratory mucus and alveolar macrophage phagocytosis, severely restrict the effectiveness of drug delivery. Mucus clearance is only evaded by particles measuring 1–5 m in size. However, most of them would be ingested by alveolar macrophages and move out via the broncho-tracheal escalator or lymphatic system [12].

Chitosan, a biocompatible and biodegradable natural polymer, has been extensively explored for administering numerous medicines, vaccines, genes and chemotherapeutic agents [13]. Various chitosan-based carriers have been studied for the pulmonary administration of various medications, such as isoniazid [14], ciprofloxacin [15], gentamicin [16] and heparin [17]. Chitosan micro/nanoparticles have been widely studied as a medication delivery vehicle for the lungs [18,19,20]. The cationic character of chitosan, due to its primary amino group, is primarily responsible for its application in various drug delivery systems [21]. Chitosan nanoparticles have been previously found to be promising nanocarriers when mixed with large carriers for pulmonary drug delivery [22]. The nanoparticles can be easily prepared by spray drying or ionic gelation techniques [23]. In the ionic gelation process, chitosan molecules containing a large number of NH_3_ groups combine with the negatively charged phosphoric ions of tri-polyphosphate (TPP) to generate crosslinked chitosan nanoparticles. Throughout the crosslinking and hardening process, water is expelled from the particles, which may aid in maintaining the drug release profile [24]. The nanoparticles, which range in size from 0.5 to 3 µm, are deposited in the alveolar region, while most of the aerosol particles are exhaled due to their smaller size [25,26,27,28,29]. Moreover, due to the cohesive nature of nanoparticles, they can achieve limited dispersed fractions when administered using a dry powder inhaler (DPI) [30]. The optimum size range for maximum drug deposition in the lung is 1 to 5 μm [31].

Hence, the nanoparticles are mostly admixed with large carriers to overcome the limited dispersed fraction from DPI [32]. Lactose is the carrier of choice in pharmaceutical aerosols due to its safety, compatibility with most drugs and approved status [33]. Different forms of lactose possess other physicochemical properties, affecting powder dispersibility from DPI and physical stability [34]. The physicochemical properties of the carrier’s particles have a significant influence on both aerosolisation and inhalation potential of DPI [22], therefore lactose microcarriers were synthesised in the suitable size range of 2–12 µm using the spray-drying method to be physically admixed with montelukast-loaded chitosan nanoparticles for potential application in the management of asthma.

Leucine is a promising excipient with multiple uses in developing spray-dried powder for inhalation. It has been discovered that adding leucine considerably improves the aerosolisation and physical stability of the generated aerosol particles. Due to its emulsifying characteristics, leucine decreases the surface tension of the aqueous feedstock. The precipitation of nanoparticles from the feedstock of chitosan and TPP solution could result in the synthesis of nanoparticles with smaller particle sizes preferred in pulmonary delivery [35]. Tween 80 can also reduce nanoparticle size by increasing chitosan’s solubility and decreasing its surface energy, inhibiting crystal formation [36]. Hyaluronic acid is one of the promising pharmaceutical compounds for pulmonary administration due to its endogenous nature, bio-adhesion and reduced macrophage phagocytosis [37,38,39]. In light of the above discussion, the current study aims to synthesise chitosan nanoparticles of variable physicochemical properties physically admixed with lactose carriers to achieve the optimum level of aerosolisation and inhalation performance when delivered using DPI.

## 2. Materials and Methods

### 2.1. The Materials

Vision pharmaceuticals, Islamabad, Pakistan, kindly donated Montelukast sodium. Chitosan (LMW, Sigma Aldrich, Burlington, MA, USA) was used as a matrix in the synthesis of nanoparticles. TPP (Sigma Aldrich, Burlington, MA, USA) was used as a crosslinker. Hyaluronic acid (Sigma Aldrich, Burlington, MA, USA) was utilised as a coating agent to prevent macrophage uptake of drug-loaded nanoparticles; Tween 80 (Sigma Aldrich, Burlington, MA, USA) and L-leucine (Sigma Aldrich, Burlington, MA, USA) were added to the nanoparticle formulation to decrease the particle size and render them hydrophobic. Lactose monohydrate (Sorbolac 400, Meggle Deutschland, Germany) was used as a carrier. All other chemicals used in the experimentation were of analytical grade.

### 2.2. Methodology

#### 2.2.1. Synthesis of Microspheres

The microspheres were synthesized using lactose as a carrier via the spray-drying technique. Spray drying allows the engineering of microparticles with desired physicochemical properties. Briefly, 2.50% of lactose solution was spray-dried using a spray dryer (Pilotech YC-015, Shanghai, China). The feed solution was pumped to a stainless-steel inner nozzle (tip diameter 0.7 mm) under optimized spray-drying conditions, i.e., an inlet air temperature of 100 °C, an outlet air temperature of 70 °C, a solution feed rate of 2 mL/min and atomizing air pressure of 5.5 bar.

The spray-dried powders thus fabricated were retrieved using a rubber spatula and placed into a 10 mL amber diagnostic vial and kept in a desiccator at room temperature (25 ± 1 °C) until further use. The percentage yield was calculated concerning the total solid content of the feed solution. The morphology of spray-dried particles was evaluated using a scanning electron microscope (SEM). The SEM photograph of each formulation was selected at suitable magnification and processed for calculation of size using processing software Image J (NIH, Bethesda, MD, USA).

#### 2.2.2. Synthesis of Montelukast-Loaded Nanoparticles

The nanoparticles were synthesised using the ionic gelation method. Briefly, 50 mg of chitosan was dissolved in 100 mL of 1.0% aqueous acetic acid at pH 5.0 to obtain a 0.5 mg/mL chitosan solution. The TPP solution was prepared using 70 mg of TPP in 100 mL of distilled water (0.7 mg/mL), and the pH was adjusted to 2 using a 1.0 M HCl solution. The TPP was added in three different concentrations to the chitosan solution with continuous stirring at 800 rpm to obtain particles of the optimum diameter. The montelukast (10 mg) was added to the TPP solution. The nanoparticles were centrifuged at 10,000 rpm for 10 min, and the particles recovered after centrifugation were re-suspended in deionised water in triplicate. After being washed, the recovered nanoparticles were gathered and freeze-dried using a freeze-dryer (Biobase, Jinan, China) [40].

#### 2.2.3. Size and Size Distribution

A Zetasizer (Nano ZS, Malvern WR14 1XZ, Worcestershire, UK) was used to evaluate the particle size and homogeneity (poly-dispersity index) of the prepared nanoparticles. The nanoparticles (3 mg) were added to 2 mL of distilled water and stirred to disperse the particles homogeneously before the analysis.

#### 2.2.4. Zeta Potential

The photon correlation spectroscopic technique was used to evaluate the charge on the surface of nanoparticles using a Zetasizer (Nano Z.S., Malvern, and Malvern WR14 1XZ, Worcestershire, UK). An aliquot of 700 µL was loaded into the folded capillary cell and assimilated with a gold electrode. The zeta potential was determined at 25 ± 1 °C with a detection angle of 90°.

#### 2.2.5. Scanning Electron Microscope

Scanning electron microscopy (Carl Zeiss Inc., Oberkochen, Germany) was used to determine the morphology of the fabricated nanoparticles. The micro- and nanoparticulate samples were placed gently on the double-sided adhesive tape mounted on an aluminium stub. The stub was tapped gently to remove excessive particles to leave a thin layer of particles on the stub surface. Representative sections were photographed at selected magnifications. The particulate samples were coated with platinum using a fine auto coater (JEOL, JEC-3000FC, Bangkok, Thailand) before examination under a microscope.

#### 2.2.6. Roughness and Circularity

Image J software was used to evaluate the roughness and circularity of particles using original SEM photographs at a specific magnification. These parameters can affect the aerosolisation and inhalational performance of the spray-dried particles. The original SEM image was converted to grey-scale (8-bit) and, subsequently, to a binary image. The plugin “analyse particle” and “roughness calculator” was run to measure the roughness (Ra) and circularity (Circ) in triplicate.

#### 2.2.7. Drug Association and Entrapment Efficiencies

The drug content and entrapment efficiency of nanoparticles were evaluated spectrophotometrically according to the reported method with some modifications [28]. Briefly, 20 mg of montelukast-loaded nanoparticles were completely dissolved in a 1% acetic acid solution, filtered using a 0.45 µm syringe filter (Aijiren, Quzhou, China) and analysed using UV visible spectrophotometrically (UV-1800, Shimadzu, India) at a ƛ maximum of 287.3 nm.

The drug content and association efficiency were determined using Equations (1) and (2).

(1) $$\mathrm{Drug}\;\mathrm{content}=\frac{\;\mathrm{Weight}\;\mathrm{of}\;\mathrm{drug}\;\mathrm{in}\;\mathrm{nanoparticles}\;}{\mathrm{Amount}\;\mathrm{of}\;\mathrm{nanoparticles}}\;\times \;100\;$$

(2) $$\mathrm{Drug}\;\mathrm{Association}\;\mathrm{Efficiency}=\frac{\mathrm{Weight}\;\mathrm{of}\;\mathrm{drug}\;\mathrm{in}\;\mathrm{nanoparticles}\;}{\mathrm{Total}\;\mathrm{weight}\;\mathrm{of}\;\mathrm{drug}\;\mathrm{added}}\;\times \;100\;$$

#### 2.2.8. Flow Characteristics

The bulk and tapped powder densities were calculated by pouring a known quantity of powder into a 5 mL measuring cylinder. The bulk of the volume was captured. The filled measuring cylinder was tapped until no further powder bed volume drop was noticed, after which a new volume was recorded (tapped volume). The bulk density and tapped density were determined by dividing the weight by the bulk or tapped volume. Using Equations (3) and (4), the Carr’s index and Hausner ratio were obtained from the values of bulk and tapped densities:

(3) $$\mathrm{Car}’s\;\mathrm{index}=\frac{\mathrm{Tapped}\;\mathrm{density}-\mathrm{Bulk}\;\mathrm{density}\;}{\mathrm{Bulk}\;\mathrm{density}}\;\times \;100\;$$

(4) $$\mathrm{Hausner}’s\;\mathrm{ratio}=\frac{\mathrm{Tapped}\;\mathrm{density}\;}{\mathrm{Bulk}\;\mathrm{density}}$$

#### 2.2.9. Differential Scanning Calorimetry (DSC)

DSC analysis of montelukast sodium and selected formulations was performed to evaluate the physical interaction of the drug with excipients. Briefly, 3 mg of each sample was taken in the heating pan of DSC (PerkinElmer, Pyres 6.0 DSC, Waltham, MA, USA) and heated over 40–300 °C at the rate of 10 °C/min [41]. The DSC thermograms of each sample were recorded in the form of exothermic and/or endothermic changes.

#### 2.2.10. Fourier Transform Infrared Spectroscopy (FTIR)

The FTIR analysis was performed to determine the possible chemical interaction between the drug and polymer or between the excipients of nanoparticle formulations. Spectra of pure montelukast sodium, TPP, chitosan, tween 80, hyaluronic acid, leucine and selected formulations were obtained using ATR-FTIR (Spectrum Two FT-IR Spectrometer, Perkin Elmer, Waltham, MA, USA).

#### 2.2.11. X-ray Diffraction Analysis (XRD)

An X-ray diffractometer (Ultima IV, Rigaku Corporation, Tokyo, Japan), with a scanning speed of 5°/min and a diffraction angle (2θ) ranging from 3° to 60°, was used to measure the crystallinity of nanoparticles. Cu- Kα radiation was used as the X-ray source at 40 kV and 30 mA.

#### 2.2.12. Drug Release

The release of the drug from the nanoparticles (F2, F4, F5 and F6) was performed in a shaking incubator (Biobase, Jinan, China) spectrophotometrically in simulated lung fluid of phosphate buffer at pH 7.4. Briefly, 10 mg of nanoparticles were loaded into an activated dialysis membrane (ZelluTrans/ROTH T4: MWCO 12,000–14,000 Da). The samples were agitated at 50 rpm, maintaining a temperature of 37 °C for 24 h. Samples of 5 mL were withdrawn at various time intervals at 0.5, 1, 2, 4, 8, 16 and 24 h. The absorbance was recorded for each sample using a UV-Visible spectrophotometer at a ƛ max of 287.3 nm. The average commutative drug released was plotted against their respective time interval [42]. Drug release was assessed using different kinetic models, where the Higuchi model was found to ideally describe the release mechanism from fabricated nanoparticles.

Qt = A √D (2C − Cs) Cst (5)

Qt represents the amount of medication released from the unit’s surface area A over time t. C represents the initial drug concentration, Cs represents the solubility of the drug in the matrix system and D represents the diffusivity of the drug in the matrix [41].

#### 2.2.13. Evaluation of Aerosolisation and Inhalation Performance

The aerodynamic diameter of the optimised formulation was evaluated using the Anderson Cascade Impactor (ACI, Copley Scientific Ltd., Nottingham, UK). All ACI components were rinsed with deionised water and then left to dry. The ACI was subsequently built from stage F to stage 0. After stage 7, a glass-fibre filter (Copley Scientific Ltd., Nottingham, UK) was installed to catch any particles that may have escaped stage 7. The stages were affixed and secured with FDA-approved silicone rubber O-rings to prevent inter-stage leakage. On stage 0, a pre-separator was mounted and linked to an induction port. The induction port and mouthpiece adapter were coupled to form an airtight seal between the Handihaler^®^ device and the induction port (Boehringer Ingelheim, Germany). As mandated in pharmacopoeias (European and American pharmacopoeias), the ACI was linked to a vacuum source (Low Capacity Pumps Models LCP5, Copley Scientific Ltd., UK) via a critical flow controller (TPK 2000, Copley Scientific Ltd., Nottingham, UK) to establish a pressure drop of 4 kPa over the DPI device [22].

The drug-loaded nanoparticles were physically admixed with spray-dried lactose microparticles in a weight-to-weight ratio of 1:9 using vortex-blending at 40 Hz for 30 min (VelpScientifica, Usmate Velate MB, Italy). Then powder mixture of 20 mg was loaded into capsules (size two), placed in the central chamber of the Handihaler^®^, pierced and the content was inhaled for 5 s at the flow rate of 48 L/min. The aerosol mass deposited on different stages of ACI was washed with 1% acetic acid solution into separate glass scintillation vials. Vials were shaken at 25 ± 1 °C for five hours in a shaker bath before being quantified for montelukast. The samples were analysed for drug content spectrophotometrically to determine various matrices demonstrating the aerodynamic behaviour of the aerosol mass.

The ACI-obtained cumulative particle size distribution functions were shown on a log probability graph. The mass median aerodynamic diameter (MMAD) was determined as the particle size corresponding to the 50th percentile on the chart. The geometric standard deviation (GSD) representing the aerodynamic particle size distribution dispersion was computed as the square root of the particle size ratio between the 84.13th and 15.87th percentiles. The emitted dosage (ED) is the total amount of montelukast collected from the mouthpiece, induction port, all stages and filter. The deposited dose (DD) was the total amount of montelukast deposited across all stages (0–7) and the filter. Percent dispersed (PD) and percent inhaled (PI) indicated the proportion of ED and DD based on the total dose (TD) as defined by Equations (6) and (7), respectively:

Percent dispersed = ED/TD × 100 (6)

Percent inhaled = DD/TD × 100(7)

The fine particle dose (FPD) was determined based on the aerosol mass deposited on the stage 3 filter, which usually represents the aerosol mass in the deep lung. As a proportion of FPD to ED and DD, respectively, the FPF and a respirable fraction (RF) were determined.

### 2.3. Statistical Analysis

The results were shown as the mean ± SD. The variation in results was considered significant when *p* ≤ 0.05, using either Student’s *t*-test (for the comparison of individual properties) or ANOVA for the comparison of multiple factors using SPSS version 20.

## 3. Results and Discussion

### 3.1. Synthesis of Chitosan Nanoparticles

The polymeric nanoparticles were successfully synthesised using the ionic gelation method. The NH_3_ group of chitosan react with the negatively charged phosphoric ions of TPP to form crosslinked chitosan nanoparticles [43]. The montelukast-loaded polymeric nanoparticles were freeze-dried to obtain solid particulate carriers for inhalation drug delivery using a dry powder inhaler. F2 with the desired chitosan-to-TPP ratio was selected as the optimum formulation based on particle morphology, higher-percentage yield, non-sticky nature and free-flowing powder compared to F1 and F3. F2 was further processed by adding Tween 80 (F4), hyaluronic acid (F5) and Leucine (F6) to the TPP solution (Table 1). The aerodynamic properties of selected formulations (F2, F4, F5 and F6) were determined after mixing with lactose microparticles as large carriers (≤10 µm).

### 3.2. Particle Size Distribution

**Figure 1:**
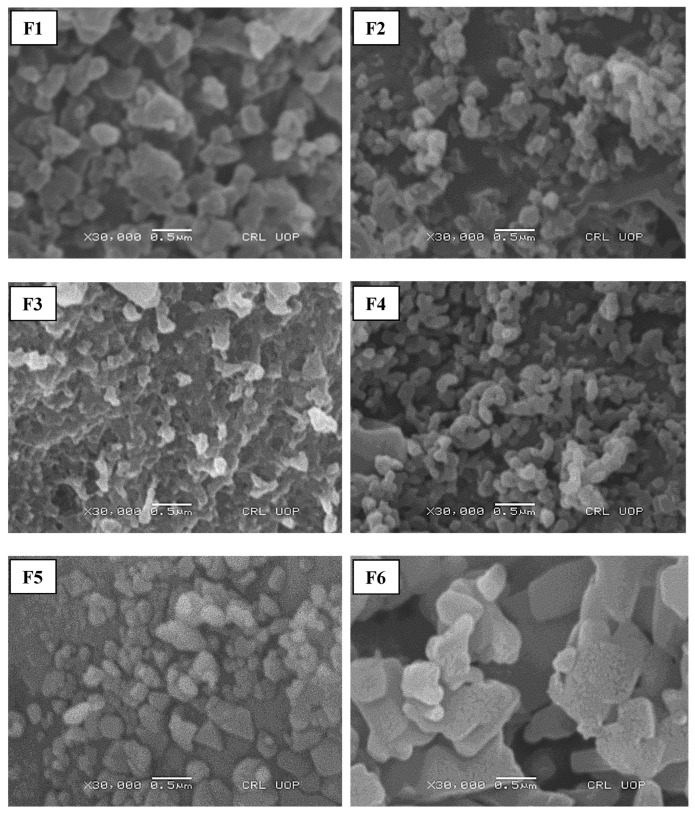
S.E.M. analysis of prepared nanoparticulate formulations.

The particle size of nanoparticles decreased with the increasing concentration of TPP in formulations (F1–F3). The reduced particle size in the case of a higher TPP-to-chitosan ratio (F3) was attributed to intermolecular solid crosslinking between chitosan molecules [44]. According to previous reports, the ratio of chitosan to TPP was crucial because it determined the size dispersion of the nanoparticles [45]. The formulation (F4) with Tween 80 added resulted in comparatively small particle size due to the presence of a surfactant that contributes to the efficient solubilisation of the polymer (chitosan), thereby leading to the production of small particles during the same stirring time when compared to its counterparts. The results suggested that Tween 80 may act as a stabilising agent during nanoparticle formation and limit crystal development by decreasing surface energy [46]. The formulations (F5) with hyaluronic acid and leucine (F6) added had a larger particle size than F2 due to the coating of nanoparticles with hyaluronic acid and leucine [32] (Table 2). The particles of F2, F4 and F5 have comparatively narrow particle size distribution, while F6 has broader particle size distribution due to the presence of leucine (Table 2; Figure 1). This limited particle size distribution could be due to an efficient interaction between positively charged chitosan and negatively charged hyaluronic acid and polyanion TPP [47]. The broader particle size distribution in the case of F6 was due to inefficient crosslinking of chitosan with TPP due to entrapped leucine in the formulation (Figure 1) [19]. The zeta potential decreased with the addition of Tween 80 in the formulation (F4) due to the non-ionic nature of Tween 80. The addition of hyaluronic acid (F5) reduced the zeta potential in comparison to F2 due to the adsorption of negatively charged hyaluronic acid on the nanoparticle surface [48]. In the case of the leucine-added formulation (F6), the surface charge (10 mV) was further reduced due to the chemical crosslinking of a free amino group of glucosamine in chitosan and conjugated L-leucine (Table 2) [19].

### 3.3. Structural Features of Nanoparticles

The morphology of initial formulations depicted that F1, F2 and F3 were comprised of spherical particles, while F4 had a bean-shaped structure. The hyaluronic-added formulation (F5) has a triangular morphology while particles of F6 with leucine added were flat and cylindrical with irregular morphology (Figure 1). The circularity of F2 was significantly higher than the other formulations with Tween 80 (F4), hyaluronic acid (F5) and leucine (F6) added, as mentioned in Table 2. Compared to different formulations, the spherical morphology of F2 could be due to the efficient crosslinking of TPP with chitosan [49]. The roughness of particles increased significantly with the addition of hyaluronic acid (F5) and leucine (F6) (*p* ≤ 0.05). This could be due to the addition of leucine, which can result in the synthesis of corrugated particles [50]. Previous studies have reported the ability of leucine to enhance the physical stability of amorphous spray-dried particles via an intermolecular interaction [51]. The addition of Tween 80 (F4) further reduced the particle size of nanoparticles by increasing the solubility of chitosan and reducing the surface energy leading to the inhibition of crystal growth [46]. The hyaluronic-acid- and leucine-added formulations (F5 and F6) have larger particle sizes concerning other formulations (F1–F4) due to the deposition of the polyanionic coating polymer on the surface of chitosan nanoparticles [52]. The roughness of particles increased with the addition of hyaluronic acid and leucine. This could be due to the reduced particle density that caused an increased particle size [53]. The roughness of the surfaces enhances the aerosolisation performance of particles [54]. It has been reported that the incorporation of leucine into the formulation (F6) before spray-drying could improve the process yield because of its anti-adherent property [55].

### 3.4. Structural Features of Physical Mixture

**Figure 2:**
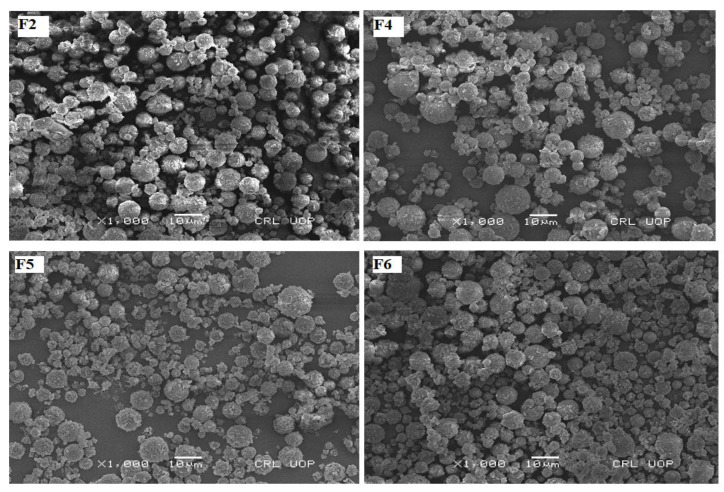
The SEM photographs of nanoparticles admixed with lactose microspheres as a carrier.

The SEM images of F2, F4, F5 and F6 physically admixed with spray-dried lactose as a large carrier are shown in Figure 2. This physical mixture of montelukast-loaded nanoparticles with spray-dried lactose microparticles depicts that nanoparticles are accumulated on spray-dried spherical lactose microparticles. Spherical-shaped particles exhibit a higher emitted dose, better flowability and a higher fine particle fraction than particles of irregular shapes in a similar particle size range [56]. The lactose monohydrate, at a concentration of 10 mg/100 mg and spray-dried in combination with leucine, produced microspheres in the range of 2 to 10 µm. The microparticle yield was 64.34% (Table 3), as mentioned in previous studies using the Pilotech spray dryer (Pilotech YC-015, Shanghai, China). The microparticles were rough, accommodating many nanoparticles on their surface (Figure 2) [57]. The bulk density of lactose was 0.245 mg/mL, while the tapped density was 0.514 mg/mL. Car’s index and Hausner’s ratio were 52.33 and 2.07, respectively (Table 3).

### 3.5. Drug Content and Entrapment Efficiency

The drug content and association efficiency were significantly higher in the case of F2 and F6 in comparison to other formulations (Student *t*-test, *p* ≤ 0.05; F2 and F6 vs. F4 and F5). This could be due to the hygroscopic nature of montelukast and the hydrophobic coating of chitosan and L-leucine that protects the drug during the synthesis of microparticles. In the case of F2, whereby particle size is significantly reduced due to the addition of Tween 80, it could also be a possible reason for the lower drug association efficiency. The formulation can achieve an optimum therapeutic level, as the inhaled drug dose is 113 times lower than oral therapy [58].

### 3.6. FTIR Analysis

**Figure 3:**
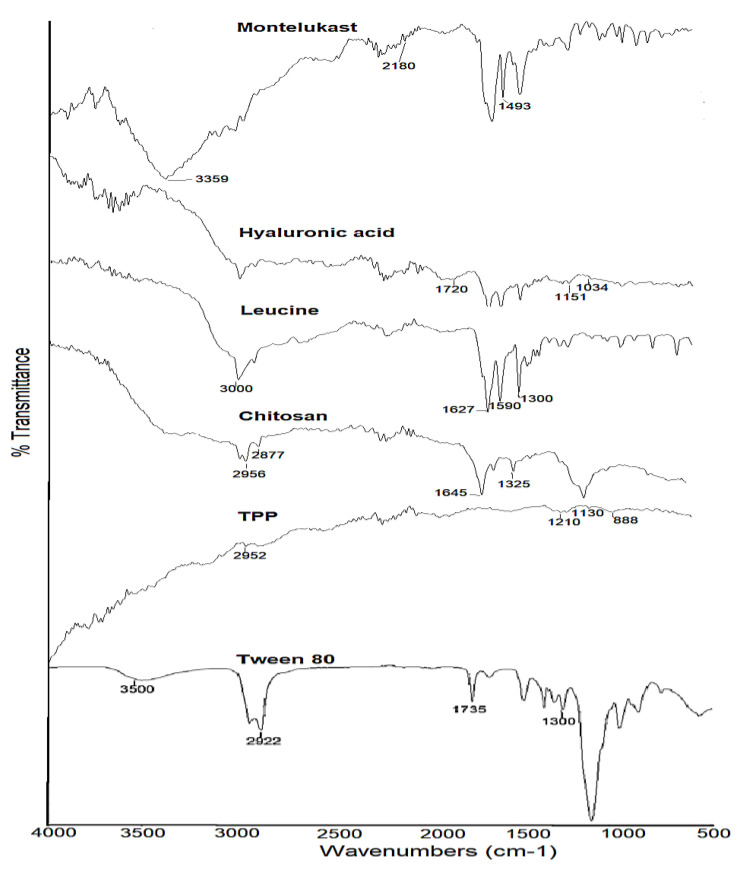
FTIR spectra of raw montelukast, chitosan, hyaluronic acid, TPP, leucine and Tween 80.

The FTIR spectra of pure montelukast sodium, chitosan, hyaluronic acid, crosslinker TPP, L-leucine, and Tween 80 are shown in Figure 3. The –OH and CO- stretching of montelukast sodium were observed at 3359 cm^−1^ and 2180 cm^−1^, respectively [25]. The 1560 cm^−1^ asymmetric stretching of the carboxylate ion and the 1493 cm^−1^ aromatic ring vibration were due to the C=C bending [59]. Chitosan exhibited absorption bands at 2956 cm^−1^ and 2877 cm^−1^, attributed to C-H symmetric and asymmetric stretching. These bands are indicative of polysaccharides [60]. The bands at approximately 1645 cm^−1^ (C=O stretching of amide I) and 1325 cm^−1^ (C-N stretching of amide I1I) revealed the presence of residual N-acetyl groups [61]. The peak at 2956 cm^−1^ was due to asymmetric CH_3_ stretching and the peak at 2925 cm^−1^ was due to asymmetric CH_2_ stretching. Similarly, at 2180 and 1582 cm^−1^, asymmetric CO stretching and NH_2_ bending vibrations are present. The peak at approximately 1408 cm^−1^ was due to carboxylic acid OH bending vibration. At 3443 cm^−1^ in the FTIR spectra of hyaluronic acid, a significant stretching band of hydroxyl groups was seen. The bands at 1720 cm^−1^ and 1648 cm^−1^ were attributed to carbonyl stretching bands of carboxylic acid and amide, respectively. The designated ether bands were located at 1151 cm^−1^ and 1034 cm^−1^, respectively [62]. The distinctive band at 1210 cm^−1^ in the spectra of TPP corresponds to the stretching vibration of P=O. The peak at 1130 cm^−1^ is attributed to symmetric and anti-symmetric stretching vibrations in the OP=O group. The band at 1090 cm^−1^ corresponds to the symmetric and anti-symmetric stretching vibrations of the PO_3_ group, whereas the peak at 888 cm^−1^ corresponds to an asymmetric stretching vibration of the POP bridge [63]. Functional groups leading to characteristic IR spectra of L-leucine include the (a) methyl group, (b) amino group and (c) carboxyl group, and OH, C=O and C-O stretching can exhibit strong IR absorption in the regions around 3000 cm^−1^, 1700 cm^−1^ and 1300 cm^−1^, respectively [64]. The methyl group characterised by aliphatic CH_3_ bending can be accounted for by the strong IR absorption bands in the region around 2800 cm^−1^–3200 cm^−1^. A strong IR band cluster often occurs in the regions 1020 cm^−1^–1250 cm^−1^ and 1590 cm^−1^–1627 cm^−1^, attributed to C-N skeletal vibration and RNH_2_ bending, respectively [65]. The FTIR spectra of Tween 80 have shown peaks at approximately 3500 cm^−1^ due to NH stretching (medium), while the peak at 2922 cm^−1^ was due to CH stretching (medium). Peaks at 2858 cm^−1^ and 1735 cm^−1^ showed CH stretching and C=O stretching.

Similarly, at 1644 cm^−1^, strong C=C and 1457 cm^−1^ CH bending was observed. OH bending and CO stretching were due to 1349 cm^−1^ and 1296 cm^−1^ peaks. Two broad bands of 2925 cm^−1^ and 2858 cm^−1^ in the FTIR spectrum of Tween 80 are attributed to the C-H stretching of the methylene group, whereas the 1735 cm^−1^ band was related to C=O stretching. The absorption band at 1460 cm^−1^ represents the C-H stretching of a methylene group, whereas the bands at 1089 cm^−1^ and 725 cm^−1^ represent the C-O-C stretching and CH_2_ rocking mode, respectively [66].

**Figure 4:**
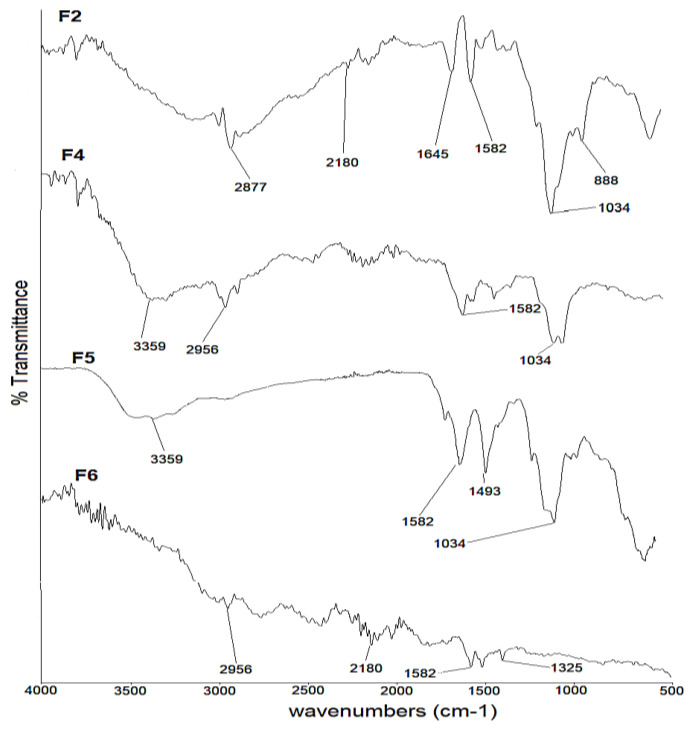
FTIR spectra of F2, F4, F5 and F6 formulations.

The FTIR spectra of selected formulations (F2, F4, F5 and F6) with the encapsulated drug are shown in Figure 4. The characteristic peaks of montelukast at 3359 cm^−1^ and 2180 cm^−1^, as well as 1560 cm^−1^ and 493 cm^−1^, are well preserved in F2, F4 and F6. In the case of F5, the spectral bands had been masked due to hyaluronic acid (Figure 4).

### 3.7. DSC Analysis

**Figure 5:**
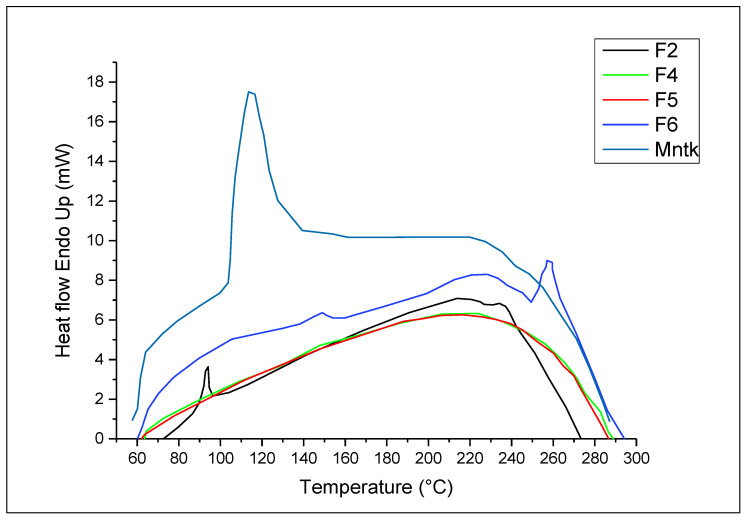
DSC analysis of pure drug and selected nanoparticles (F2–F4) formulations.

The DSC thermogram of pure montelukast sodium was melted within 60–100 °C (Figure 5). The peak was broader due to the amorphous nature of the drug [67]. In the case of F2, the drug showed a sharp endothermic peak at 90 °C, possibly contributing to the recrystallisation of the drug after lyophilisation. In the case of F4 and F5, whereby Tween 80 and hyaluronic acid were added, respectively, no sharp peak of melting endotherms was observed. The disappearance of the endothermic melting peak of the drug was due to dehydration of hyaluronic acid at 101 °C, whereby the two peaks intermingle with each other. The loss of moisture (dehydration) at 101 °C for hyaluronic was also observed in previous studies [68]. The endothermic peak of the melting drug in the case of F6 was delayed and appeared at 145 °C due to encapsulation of the drug in chitosan with hydrophobic coating from leucine.

### 3.8. XRD Analysis

**Figure 6:**
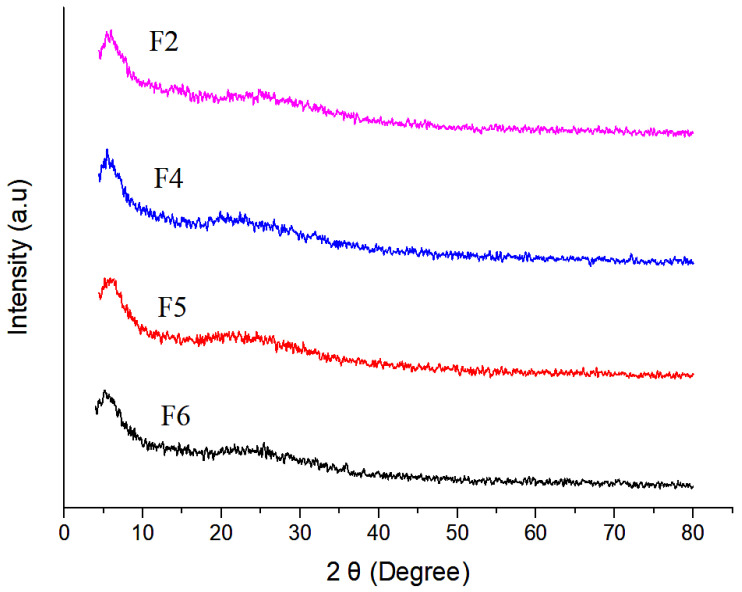
XRD analysis of selected nanoparticulate formulations.

The XRD pattern of chitosan nanoparticles (F2–F6) is shown in Figure 6. The formation of chitosan nanoparticles was confirmed by XRD analysis, where polymer chains of chitosan crosslinked with each other by TPP. The absence of sharp peaks in the XRD diffractogram depicts the amorphous nature of synthesised nanoparticles. However, the two characteristic peaks at 2 Theta of 5° and 18° were due to the crystalline nature of the drug in the nanoparticles [57].

### 3.9. Release Profile

**Figure 7:**
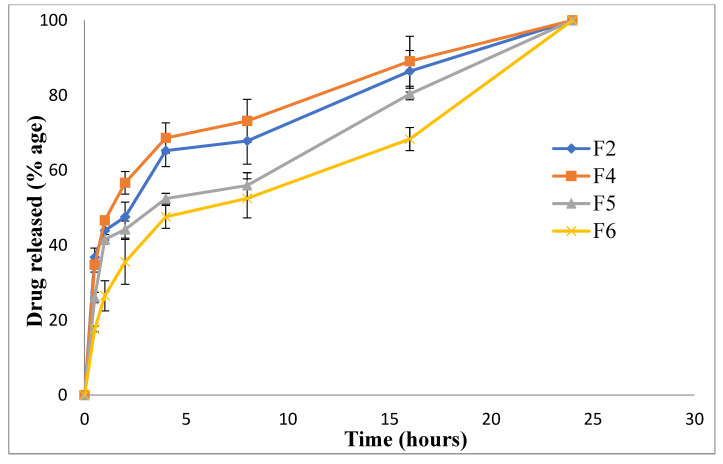
Drug release profile of nanoparticulate formulations.

The release of drugs from nanoparticles depicts a higher-burst release in the case of F2 and F4 compared to F5 and F6. This was due to the small particle size (Table 2; Figure 1), thereby increasing the surface area exposed to the dissolution medium. The initial burst release in the case of F2 and F4 was due to the free drug on the surface of nanoparticles, followed by a controlled release pattern due to the diffusion of encapsulated montelukast from nanoparticles into the release medium [69]. In F5 and F6, the formulations were coated with hyaluronic acid and L-Leucine, respectively, so their release was comparatively more retarded (Figure 7). The steady release was advantageous for prolonging the therapeutic impact [41]. Four in vitro release models, including zero order, first order, the Higuchi model, and the Korsmeyer–Peppas model, were used to examine the release mechanisms of selected formulations (F2, F4, F5 and F6). In our analysis, the release kinetics fit the Higuchi model with an R2 of less than 0.90, indicating that the active release of the drug from the dosage form follows Fick’s law of diffusion (Table 4). As illustrated in previous studies, the Higuchi model is ideal for describing the drug dissolution profile of chitosan nanoparticles [69].

### 3.10. Aerosolisation and Inhalation Performance

The aerodynamic performance of powder comprised of nanoparticles physically admixed with spray-dried lactose microspheres when delivered using Handihaler and evaluated using ACI is shown in Table 5. The MMAD of the powder mixture in the range of 1–2 µm was the most suitable size range for deep lung inhalation [70]. However, the aerodynamic particle size (GSD) distribution was significantly higher in the case of F6 when compared to other formulations (*p* ≤ 0.05). The higher GSD in the case of F6 could be attributed to the large particle size, lower roughness and circularity of nanoparticles (F6) mixed with large carriers (Table 2 and Table 5). When a total dose of montelukast sodium was administered in the range of 0.23 to 0.57 mg, it was found that F6 achieved the highest percent dispersed form DPI (Handihaler), and the inhaled fraction was significantly higher than in other formulations. This suggests that leucine, due to its hydrophobic properties, increases the loosely attached particles to microspheres, acting as glidants to improve the flowability of powder [71]. The FPD in the range of 0.12–0.32 mg is therapeutically sufficient to relieve symptoms of asthmatic patients. Overall, due to lower MMAD, F6 was a successful formulation that achieved significantly higher FPF and therapeutic efficacy.

## 4. Conclusions

Chitosan nanoparticles were synthesised by the ionic gelation method. The nanoparticles’ size ranged from 220 to 380 nm, with a relatively homogeneous size distribution (PDI ≤ 0.50). The nanoparticles have positive zeta potential (11 to 22 mV), resulting in improved interaction with the negatively mucosal surfaces of lung parenchyma. The roughness and circularity of nanoparticles can affect the aerodynamic performance of aerosol powder either due to the interaction with course carrier particles or their mutual interaction when used as DPI powder without carriers. The circularity of simple chitosan nanoparticles (F2) was higher than other formulations, while the roughness of hyaluronic-acid- and leucine-added nanoparticles (F5 and F6) was significantly higher than F2 and F4. The drug content of nanoparticles was optimum concerning the inhaled dose of montelukast, and the release of the drug was sustained after an initial burst drug release up to 20–40%. Due to the agglomeration tendency of nanoparticles, they were mixed with lactose microspheres (5.60 ± 2.37 µm) prepared by the spray-drying method and evaluated for aerodynamic performance using ACI. The DPI formulations achieved a dispersed fraction of 75 to 90% within the aerodynamic particle size range of 1–2 µm. The fine particle fraction (FPF) of 28–83% depicts that delivering montelukast in the form of DPI could be an effective strategy for potential application in the management of asthmatic conditions.

## Figures and Tables

**Table 1:** Formulation of montelukast-loaded chitosan nanoparticles.

S. No	Chitosan (% *w*/*w*)	TPP (% *w*/*w*)	Montelukast (% *w*/*w*)	Tween 80 (% *w*/*w*)	Hyaluronic Acid (% *w*/*w*)	Leucine (% *w*/*w*)
F1	54.054	18.918	27.027	0.000	0.000	0.000
F2	45.454	31.818	22.727	0.000	0.000	0.000
F3	39.215	41.176	19.607	0.000	0.000	0.000
F4	45.443	31.810	22.721	0.025	0.000	0.000
F5	40.816	28.571	20.408	0.000	10.208	0.000
F6	40.816	28.571	20.408	0.000	0.000	10.208

**Table 2:** Physicochemical characteristics of montelukast-loaded chitosan nanoparticles.

Parameters	F2	F4	F5	F6
Size (nm)	256.71 ± 11.23	220.56 ± 15.23	276.22 ± 08.23	382.88 ± 17.23
PDI	0.307	0.357	0.397	0.416
Zeta potential (mV)	22.23	18.06	14.10	11.40
Roughness (nm)	5.07 ± 0.06	5.9 ± 0.04	12.402 ± 0.49	8.12 ± 0.29
Circularity	0.880 ± 0.07	0.721 ± 0.05	0.673 ± 0.05	0.683 ± 0.07
Drug content (µg/mg)	56.84	31.90	22.78	42.95
Association efficiency (%)	73.85	51.04	45.56	85.90
Yield (%)	55.02 ± 2.77	67.09 ± 3.23	70.07 ± 3.15	72.03 ± 3.97

**Table 3:** Physicochemical characteristics of spray-dried lactose microparticles.

Physicochemical Properties	
Particle size distribution (µm)	5.60 ± 2.37
Bulk density (g/mL)	0.25 ± 0.07
Tapped density (g/mL)	0.52 ± 0.04
Cars index	52.33 ± 1.94
Hausner’s ratio	2.07 ± 0.14
Yield (%)	64.34 ± 5.24

**Table 4:** Release kinetics of drug from nanoparticle formulations.

Formulations	Zero-Order Kinetics	First-Order Kinetics	Higuchi Model	Korsmeyer-Peppas Model
F2	0.751	0.911	0.941	0.778
F4	0.774	0.972	0.952	0.763
F5	0.815	0.981	0.965	0.795
F6	0.740	0.933	0.932	0.751

**Table 5:** Aerosolisation and inhalation profile of chitosan nanoparticles admixed with spray-dried lactose microparticles.

	F2	F4	F5	F6
Mass median aerodynamic diameter (µm)	1.23	2.34	1.78	1.34
Geometric standard deviation	2.95	2.91	2.92	4.78
Total dose (mg)	0.57	0.31	0.23	0.43
Emitted dose (mg)	0.43	0.27	0.20	0.38
Deposited dose (mg)	0.12	0.17	0.13	0.32
Per cent dispersed (%)	75.12	85.75	86.55	89.12
Per cent inhaled (%)	21.37	55.35	55.33	74.11
Fine particle dose (mg)	0.12	0.17	0.13	0.32
Fine particle fraction (%)	28.45	64.55	63.92	83.22
Respirable fraction (%)	100	93.44	100	100

## Data Availability

The data presented in this study are available on request from the corresponding author.
